# Unbiased approach to identify and assess efficacy of human SARS-CoV-2 neutralizing antibodies

**DOI:** 10.1038/s41598-022-19780-7

**Published:** 2022-09-15

**Authors:** Xia Cao, Junki Maruyama, Heyue Zhou, Yanwen Fu, Lisa Kerwin, Colin Powers, Rachel A. Sattler, John T. Manning, Alok Singh, Reyna Lim, Laura D. Healy, Sachi Johnson, Elizabeth Paz Cabral, Donghui Li, Lucy Lu, Arthur Ledesma, Daniel Lee, Susan Richards, Laura Rivero-Nava, Yan Li, Weiqun Shen, Karen Stegman, Benjamin Blair, Shinji Urata, Magumi Kishimoto-Urata, Jamie Ko, Na Du, Kyndal Morais, Kate Lawrence, Ianne Rivera, Chin-I. Pai, Damien Bresson, Mark Brunswick, Yanliang Zhang, Henry Ji, Slobodan Paessler, Robert D. Allen

**Affiliations:** 1 Sorrento Therapeutics, Inc., San Diego, CA 92121 USA; 2 Galveston National Laboratory, Department of Pathology, University of Texas Medical Branch, Galveston, TX 77555 USA

**Keywords:** Viral infection, Drug development

## Abstract

Coronavirus disease 2019 (COVID-19) continues to significantly impact the global population, thus countermeasure platforms that enable rapid development of therapeutics against variants of SARS-CoV-2 are essential. We report use of a phage display human antibody library approach to rapidly identify neutralizing antibodies (nAbs) against SARS-CoV-2. We demonstrate the binding and neutralization capability of two nAbs, STI-2020 and STI-5041, against the SARS-CoV-2 WA-1 strain as well as the Alpha and Beta variants. STI-2020 and STI-5041 were protective when administered intravenously or intranasally in the golden (Syrian) hamster model of COVID-19 challenged with the WA-1 strain or Beta variant. The ability to administer nAbs intravenously and intranasally may have important therapeutic implications and Phase 1 healthy subjects clinical trials are ongoing.

## Introduction

Since the first reports in December of 2019, the severe acute respiratory disease syndrome coronavirus 2 (SARS-CoV-2) has caused a global pandemic and has continued to significantly impact the health and lives of the human population.

In early COVID-19 disease, intravenous administration of neutralizing antibodies (nAbs) is an effective means of lessening progression and overall severity of disease^1,2^. As COVID-19 is a predominantly respiratory disease, exploration of alternative modes of antibody administration including intranasal delivery, in contrast to the standard intravenous modes, may provide an expedient means of delivering antibodies and increasing the lung bioavailability of anti-COVID-19 nAbs as well as augmenting the developing host-directed immune response, preventing exacerbation of clinical symptoms and hospitalization^3^.

To date, public health agencies have combatted infections leading to COVID-19 by relying heavily on quarantine, social distancing, vaccination, and countermeasure strategies. Despite these efforts, the continued spread of SARS-CoV-2 has led to the emergence of several Variants of Concern (VOCs) that have risen in prevalence worldwide^4^. The emergence of VOCs, exemplified by the Alpha and Beta variants, demonstrates the need for countermeasure platforms that enable rapid development of therapeutics to treat populations impacted by pandemic threat pathogens.

The Sorrento G-MAB™ library^5^ utilizes a single chain variable fragment (scFv) antibody phage display library constructed from the antibody repertoire of over 600 healthy individuals, encoding the donor paratopes as well as an expanded novel collection of antigen recognition regions created by random pairings of heavy and light chain-encoding genes from within the donor pool. Selection of nAbs within phage display human antibody libraries also allows for functional, unbiased selection of Spike-binding nAbs independent of the selective pressures underlying natural immunogenicity^6–8^. The identified nAbs can be characterized and tested for virus neutralizing potency without reliance on identification of immunoglobulin coding sequences from survivor patient populations or immunization of transgenic animals. The G-MAB system provides a means of pre-banking a safe and potent collection of nAbs with the capacity to treat pandemic threat pathogens as they emerge over time.

Of note, it has been shown in the context of multiple virus infections that virus-specific antibodies can lead to exacerbation of disease symptoms through a process termed antibody dependent enhancement (ADE)^9,10^. To reduce the risk of ADE resulting from administration of G-MAB-derived nAbs, the IgG1 Fc regions were modified by introducing specific amino acid substitutions (L234A, L235A [LALA])^11^. The LALA Fc modification reduces binding affinity to the Fcγ receptors while providing a similar blockade to interactions between SARS-COV-2 and the angiotensin-converting enzyme 2 (ACE2) receptor expressed on susceptible cells in the lung and other organs^12–14^. The LALA nAbs were formulated for IV or IN administration and profiled for protective efficacy using established small animal models of virus pathogenesis^3^.

Data presented herein demonstrates the identification, in vitro binding and neutralizing activity of two G-MAB nAbs, STI-2020 and STI-5041, against the SARS-CoV-2 WA-1 strain as well as the Alpha and Beta VOCs. Additionally, we describe the protective effects of STI-2020 and STI-5041 administered IV or IN in the golden (Syrian) hamster model of COVID-19 disease following challenge with either the WA-1 strain or Beta SARS-CoV-2 VOC.

## Results

### Rapid discovery and characterization of neutralizing antibodies

**Figure 1:**
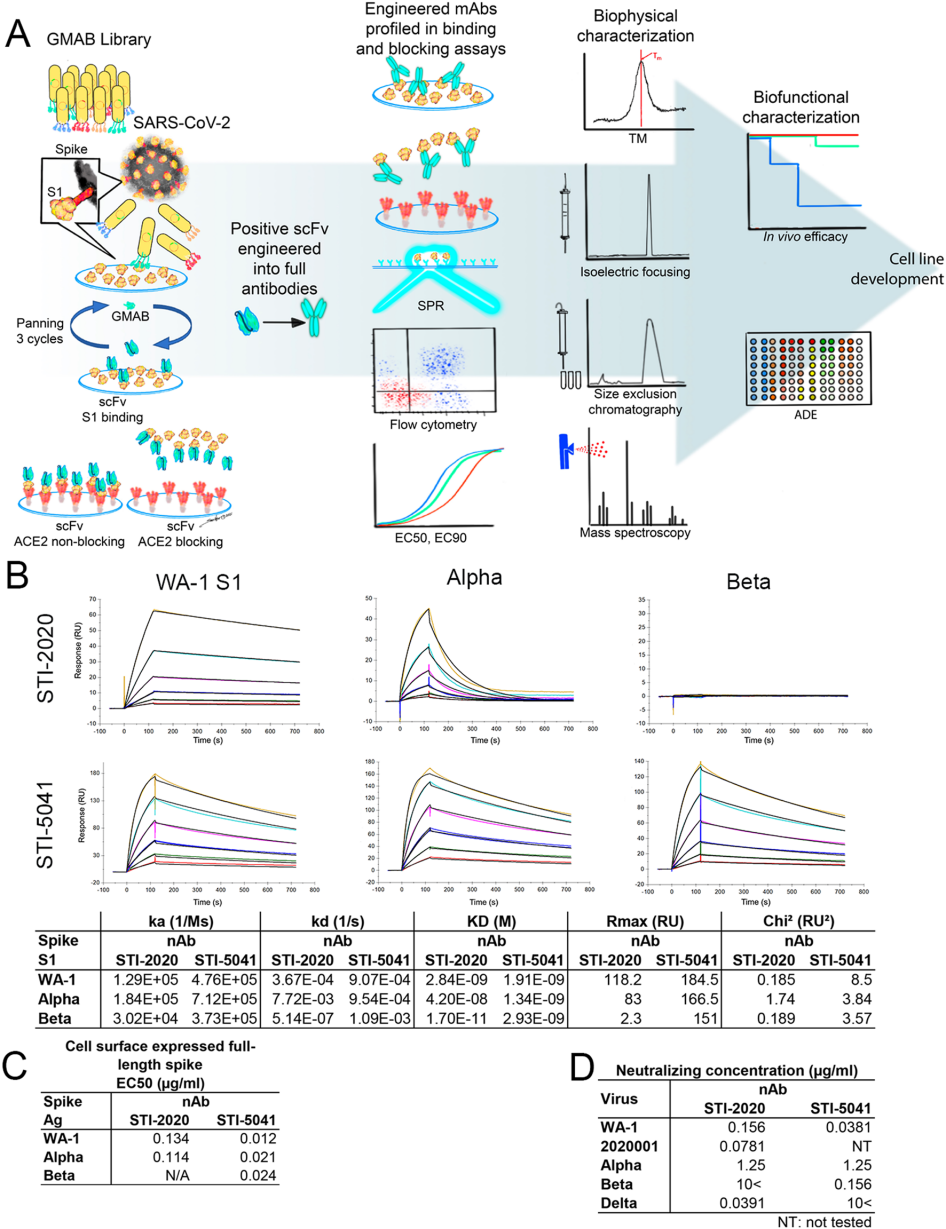
Rapid Discovery of Neutralizing Antibodies. (**A**) The G-MAB phage display library was panned for SARS-CoV-2 Spike S1 subunit-binding scFv fragments. Following confirmation of binding activity and blocking of S1:ACE2 interactions by candidate scFvs, the most potent of these candidates were converted to IgG1 antibodies bearing the LALA Fc modification. Candidate nAbs were characterized for binding of Spike S1 subunit and neutralization of related clinical SARS-CoV-2 isolates. Affinity maturation of potent nAbs was carried out in parallel to biophysical profiling, cell line development, and evaluation of protective efficacy for the parental nAbs, S1D2 and S7E3 to yield STI-2020 and STI-5041, respectively. Artwork credit: William SooHoo. (**B**) Affinity measurements of STI-2020 and STI-5041 for Spike S1 domain from the following isolates and VOCs: USA/WA-1/2020(WA-1) isolate, D614G 2020001 isolate, B.1.1.7 VOC (Alpha), B.1.351 VOC (Beta). The antibody affinities were measured using SPR on a BIAcore T200 instrument using a 1:1 binding model. (**C**) Spike protein derived from the WA-1 and 2020001 (D614G) SARS-CoV-2 isolates were independently expressed on the surface of HEK 293 cells. Serially-diluted STI-2020 or STI-5041 were assayed for Spike protein binding by flow cytometry. To quantify antibody binding, mean fluorescent intensity was measured for each dilution tested and the EC_50_ value was calculated for each nAb. Representative replicate experiments are shown. (**D**) STI-2020 and STI-5041 were evaluated in neutralizing test for potency against SARS-CoV-2 USA/WA-1/2020, 2020001 (D614G), B.1.1.7 VOC (Alpha), B.1.351 VOC (Beta) and B.1.617.2 (Delta).

To determine candidate anti-SARS-CoV-2 neutralizing antibodies (nAbs) for further screening, Sorrento’s G-MAB™ library (scFv antibody phage library) was used^5^. Antibodies were screened for binding to a recombinant His-tagged SARS-CoV-2 Spike S1 subunit, selecting for candidates with high affinity to the target antigen (Fig. 1A). Following confirmation of S1 binding by ELISA, clonal scFv preparations were tested by competition ELISA for disruption of Spike S1:ACE2 binding. Candidate scFvs with high S1 binding affinity and/or the capacity to block Spike S1:ACE2 binding were converted into and subsequently expressed as full length human IgG1-LALA Fc antibodies. A nAb candidate termed STI-1499 displayed potent SARS-CoV-2 superior neutralizing activity, wherein affinity maturation was undertaken and identified the most potent affinity matured clone, STI-2020. Similarly, STI-5041was derived from a distinct G-MAB clone, S7E3, which bound with high affinity to SARS-CoV-2 spike. Both STI-2020 and STI-5041 were further profiled for binding affinity using surface plasmon resonance (SPR). STI-2020 bound to the S1 region of the Spike protein of the USA/WA-1/2020 (WA-1) isolate with an affinity of 2.84 nM and 42.0 nM to the Alpha variant, and binding to the Beta variant was not observed (Fig. 1B). The affinity of STI-5041 was determined to be 1.91 nM to the WA-1 isolate, 1.34 nM to the Alpha variant, and 2.93 nM to the Beta variant, demonstrating potent affinity to clinically meaningful variant Spike proteins. Extending these studies, STI-2020 and STI-5041 were tested for the binding of full-length Spike protein derived from naturally emerging viruses expressed on a cell surface. These binding studies demonstrated STI-2020 bound the WA-1 isolate and Alpha variant with similar affinity, whereas binding affinity to the Beta variant was undetectable in the assay. In contrast, STI-5041 bound the WA-1 isolate with an EC50 of 0.012 µg/mL and exhibited similar affinity for both the Alpha and Beta variants, 0.021 and 0.024 µg/mL, respectively (Fig. 1C). Using the virus neutralization assay, the neutralizing activity of STI-2020 and STI-5041 was tested against WA-1, an early variant isolate encoding the Asp (D) to Gly (G) mutation at amino acid residue 614 of the Spike protein (2020001), Alpha and Beta variants. Again, we observed that STI-2020 neutralized all isolates tested except the Beta variant, whereas STI-5041 retained neutralizing activity across the isolates tested except the Delta variant (Fig. 1D).

### Bioavailability and pharmacokinetics of neutralizing antibodies

**Figure 2:**
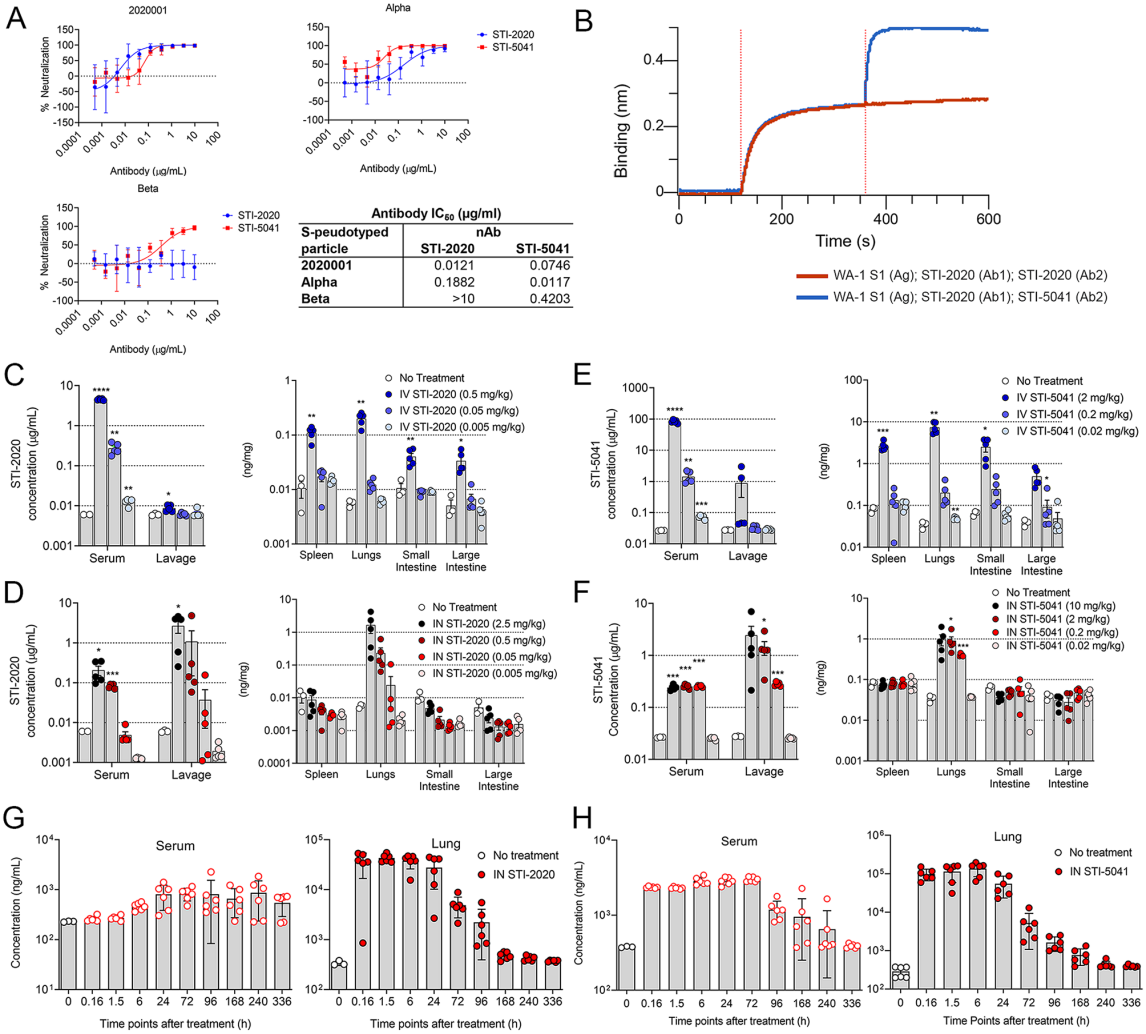
Pharmacokinetic and bioavailability of Neutralizing Antibody. (**A**) Neutralization of SARS-CoV-2 Spike-pseudotyped VSV by STI-2020 and STI-5041. VSVΔG-luciferase was pseudotyped with the indicated spike variant, incubated with STI-2020 or STI-5041 at a range of 0.0005–10 µg/mL for 30 min, then added to 293-ACE2 target cells. Absolute IC_50_ was calculated from luciferase values and are indicated. Experiments were performed at least three independent times and data presented as the mean ± SD. (**B**) Epitope binning was performed as described in the Materials and Methods. The sensorgram shows STI-5041 can bind to SARS-CoV-2 S1 and STI-2020 complex (blue line) and indicates that STI-5041 and STI-2020 bind to distinct epitopes. (**C–H**) *Biodistribution:* Concentration of STI-2020 (**C**,**D**) or 5041 (**E**,**F**) in serum and lung lavage or lysates of spleens, lungs, small intestines, and large intestines collected from female CD-1 mice administered STI-2020 IV at doses of 0.5 mg/kg (Dark blue circle), 0.05 mg/kg (Sky blue circle), or 0.005 mg/kg (light blue circle) or IN at doses of 2.5 mg/kg (black circle), 0.5 mg/kg (Maroon circle), 0.05 mg/kg (Red Circle), and 0.005 mg/kg (Rose circle) or STI-5041 administered IV at doses of 2 mg/kg (Dark blue circle), 0.2 mg/kg (Sky blue circle), and 0.02 mg/kg (light blue circle) or IN at doses of 10 mg/kg (Black circle), 2 mg/kg (Maroon circle), 0.2 mg/kg (Red circle), and 0.02 mg/kg (Rose circle) at 24 h post-administration as compared to samples collected from untreated mice. Values represent mean ± SEM (n = 3 animals no treatment group, n = 5 in treatment groups). Significant differences are denoted by * *p* < 0.05; ** *p* < 0.01; *** *p* < 0.001, **** *p* < 0.0001. *Pharmacokinetics:* Concentration of STI-2020 (**G**) or STI-5041 (**H**) in lungs and isolated serum collected from female CD-1 mice administered STI-2020 or STI-5041 intranasally (IN) at a dose of 5 mg/kg or 20 mg/kg, respectively. Samples from treated mice were collected at the indicated timepoint post-administration; antibody concentrations were quantified by ELISA and compared to samples collected from untreated mice. Overlay of antibody concentrations in lung tissue vs. serum following IN administration of a 5 mg/kg or 20 mg/kg dose. Values represent mean ± SD (n = 3 animals no treatment group, n = 6 per time point in treatment groups).

Pseudotyped virus was used to determine the IC_50_ of the nAbs STI-2020 and STI-5041. The IC_50_ for Alpha- and Beta-based pseudotypes was within tenfold of that measured in assays with the 2020001 D614G pseudotype for each nAb, with the exception of the STI-2020 IC_50_ against the Beta variant, which was estimated to be > 10 µg/mL (Fig. 2A). Epitope binning with coordinated binding against the WA-1 RBD demonstrated distinct epitope binding of STI-2020 versus STI-5041, which may in part explain the lack of neutralizing activity seen for STI-2020 against the Beta variant (Fig. 2B). The biodistribution of STI-2020 and STI-5041 was evaluated following delivery by either the intravenous or intranasal route. These studies illustrate the potential effects of delivery route on the timing of antibody exposure in the lung tissue and blood of treated mice. Following IV treatment at a dose level of 0.5 mg/kg, STI-2020 was detected in the serum, spleen, lungs, small intestine, and large intestine. Detected levels in the serum following the 0.5 mg/kg dose averaged 4.5 µg/mL, while STI-2020 was present at average concentrations less than 0.01 µg/mL in lung lavage material at each of the IV doses tested (Fig. 2C, upper left panel). Upon processing of lung tissue, antibody was detected at a mean concentration of 0.2 ng/mg of tissue in the 0.5 mg/kg IV dose group. Lower IV doses of STI-2020 did not lead to a statistically significant detection in lung tissue. Antibody levels in the spleen reached a similar average concentration at 24 h to that seen in lung tissue (0.1 vs. 0.2 ng/mg of tissue, respectively). Similarly, antibody was detectable in both the small and large intestines only at the highest dose level, with average concentrations of 0.04 and 0.03 ng/mg of tissue, respectively (Fig. 2C, upper right panel).

Following intranasal (IN) administration of STI-2020, the concentration of antibody in the serum at 24 h reached an average value of 0.21 µg/mL in the 2.5 mg/kg dose group and 0.08 µg/mL in the 0.5 mg/kg dose group. As compared to IV treated animals at the 0.5 mg/kg dose, STI-2020 administered IN resulted in a thirtyfold lower concentration of antibody in serum. In contrast, STI-2020 concentrations in lung lavage samples following IN dosing reached average concentrations of 2.7 µg/mL in the 2.5 mg/kg dose group and 1.1 µg/mL in the 0.5 mg/kg group. In 4 of 5 treated mice, levels of STI-2020 in lung lavage following IN administration of 0.5 mg/kg were elevated 6 to 37-fold over those observed in lung lavage following an equivalent IV dose. In lung tissue samples 24 h following the 0.5 mg/kg IN dose, STI-2020 was detected at concentrations similar to those recorded in IV-treated animals at the same dose level. At the higher dose of 2.5 mg/kg IN, the average concentration in the lung was measured at 1.66 ng/mg of tissue. STI-2020 levels in spleen, small and large intestine at all IN dose levels tested did not rise to levels above background (Fig. 2D).

The biodistribution of STI-5041 when administered IV at 2 mg/kg demonstrated the antibody was detectable in the serum, spleen, lungs, small and large intestine. At 2 mg/kg STI-5041, 82.9 µg/mL in the serum was observed, as well as 2.6 ng/mg in the spleen, 7.3 ng/mg in the lungs, 2.5 ng/mg in the small intestine and 0.5 ng/mg in the large intestine. At the 0.2 mg/kg IV dose of STI-5041, 1.4 µg/mL was measured in the serum, whereas all other concentrations of STI-5041 (0.2 mg/kg or 0.02 mg/kg) were below 1 µg/mL in fluid or 1 ng/mg in tissue of organs measured (Fig. 2E). STI-5041 was then administered IN at 10 mg/kg, 2 mg/kg, 0.2 mg/kg and 0.02 mg/kg. At the IN doses of 10, 2, and 0.2 mg/kg, STI-5041 was detected in the serum, the lung lavage and lung tissue. The serum concentration measured at the three doses of STI-5041 were all similar, whereas in the lung lavage a dose response from 2.4 μg/mL down to 0.28 µg/mL was observed. The concentrations detected in the lung tissue were 0.968 ng/mg, 0.905 ng/mg and 0.4 ng/mg for 10 mg/kg, 2 mg/kg and 0.2 mg/kg, respectively (Fig. 2F). Overall, IN delivery of either STI-2020 or STI-5041 led to lower serum concentrations, higher lavage concentrations, and similar tissue concentrations when compared to IV delivery. This overall suggests that the IN administration serves to increase the amount of antibody in the pulmonary lavage material, allowing for potentially faster neutralization of respiratory viral particles in the extravascular pulmonary space.To characterize STI-2020 pharmacokinetics following intranasal dosing at 5 mg/kg, antibody levels in CD-1 mouse lung tissue lysates and serum were quantified at designated timepoints spanning a total of 336 h using a human antibody detection ELISA. There were no quantifiable concentrations of STI-2020 antibody in the pre-dose samples. Following IN administration of STI-2020, the antibody concentration was quantifiable up to 240 and 336 h in the lungs and serum, respectively (Fig. 2G). We observed a mouse-to-mouse variability at each time point that could be inherent to the delivery method^15^. Average antibody concentration in the lung measured 10 min after dosing was nearly 70 percent of the maximum antibody concentration (C_max_) measured during the experiment. The C_max_ value of STI-2020 in the lungs was measured at 1.5 h post-administration at a value of 43,073.2 ng/mL. In the lungs, an apparent terminal half-life (T_1/2_) of 32.21 h was measured when analyzed between 0.15 and 240 h. Under these conditions the R^2^ value equaled 0.932, however when the data were analyzed between 0.15 and 168 h the R^2^ value increased to 0.987 but the T_1/2_ dropped to 25.07 h for the lung samples. Kinetics of STI-2020 exposure in the lungs following intranasal administration was accompanied by a slower kinetic of detectable antibody in the serum of treated mice (Fig. 2G). Antibody was first detected in the serum at 6 h post-administration and the C_max_ of 871 ng/mL was reached at the 240 h timepoint (T_max_). Serum antibody concentrations were within 90% of the recorded C_max_ by the 24 h timepoint. Antibody levels remained relatively constant in serum over the period spanning 24–240 h, which is in keeping with the calculated STI-2020 serum half-life observed following IV administration of 240 h in mice (data not shown). The total systemic exposure (AUC_last_) was significantly higher in the lungs than in the serum of treated mice (AUC_last_ were 1,861,645.8 and 248,675.5h* ng/mL respectively).

STI-5041 was administered IN at 20 mg/kg and the PK parameters were assessed (Fig. 2H, lower panels). In the serum, the time observed to reach C_max_ (T_max_) was 72 h, a C_max_ of 2,995.04 ng/mL, a T_1/2_ of 116.5 h, the AUC_last_ was 441,158.6h* ng/mL with an R^2^ value of 0.904. In contrast, the same dose of STI-5041 in the lung resulted in a T_max_ of 6 h, a C_max_ of 136,095.9 ng/mL, a terminal half-life of 15.2 h, AUC_last_ was 4,120,508.6h* ng/mL with an R^2^ value of 0.987.

### Treatment using IV administered STI-2020 or STI-5041 in the golden (Syrian) Hamster model of COVID-19

**Figure 3:**
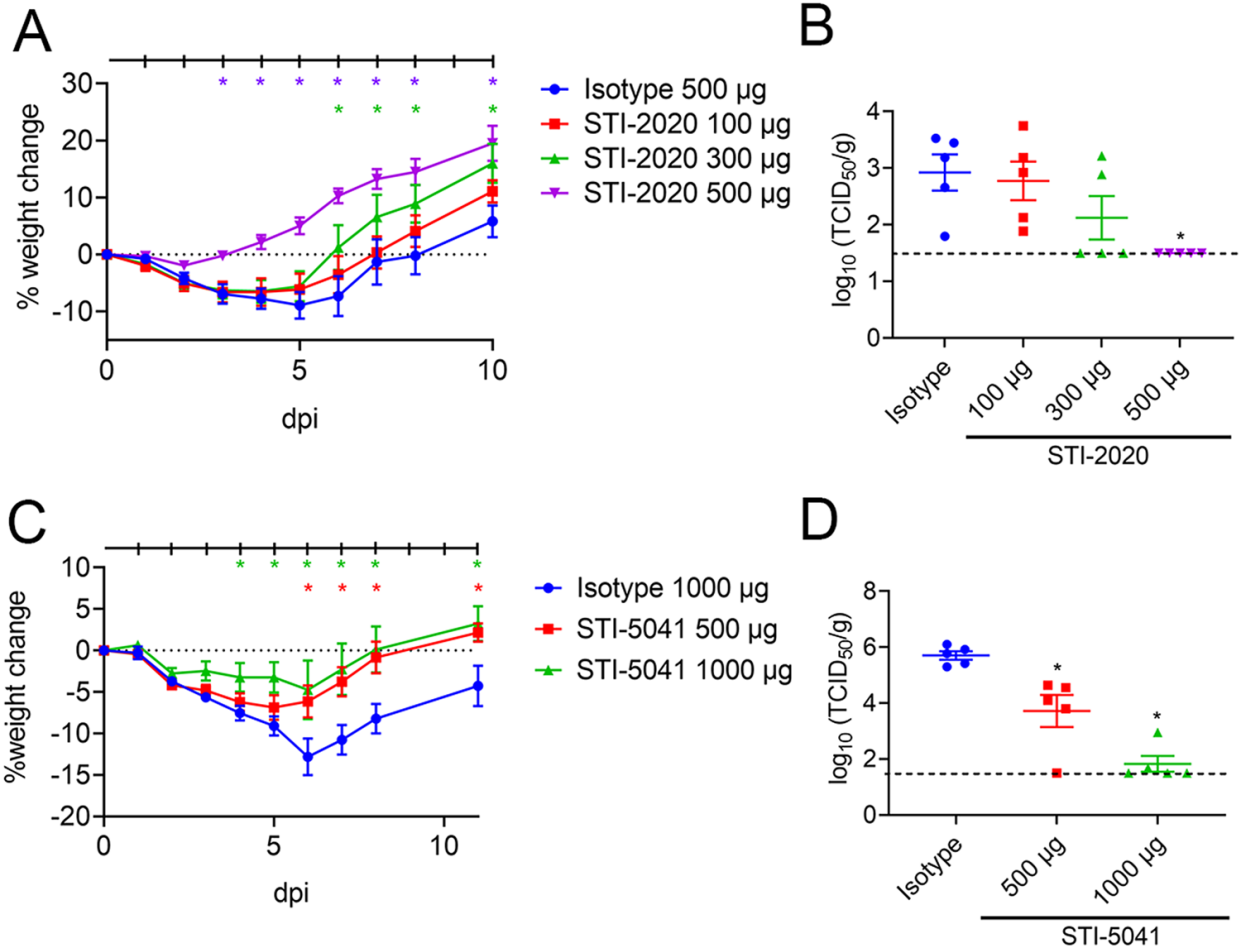
Efficacy of Intravenous (IV) Delivery of Neutralizing Antibodies in the golden (Syrian) Hamster Model of COVID-19. Female hamsters were inoculated with SARS-CoV-2 USA/WA-1/2020 (**A**,**B**) or SARS-CoV-2 Beta variant (**C**,**D**) on day 0. One-hour post-infection, animals were administered a single intravenous dose of Isotype control IgG (500 µg) or, For A and B, STI-2020 (100 µg, 300 µg, or 500 µg); For C and D, Isotype control IgG (1,000 µg) or STI-5041 (500 µg, or 1,000 µg). Weight changes were recorded for up to 11 days. (**A**) Average % weight change ± SEM was plotted for each group. Days in which there was a significant difference in average % weight change compared to Isotype control IgG 500 µg-treated animals are denoted by * (*p*-value < 0.05). (**B**) Lung tissues collected from five animals per group and virus titers were determined on day 5. A broken line indicates the detection limit of the assay (< 1.5 TCID_50_/g). (**C**) Average % weight change ± SEM was plotted for each group. Days in which there was a significant difference in average % weight change for STI-5041 at 500 µg or 1,000 µg compared to Isotype control IgG 1000 µg-treated animals are denoted by * (*p*-value < 0.05). (**D**) Lung tissues collected from five animals per group administered Isotype control IgG (1,000 µg) or STI-5041 (500 µg or 1,000 µg) and virus titers were determined on day 5.

SARS-CoV-2 pathogenesis in the golden (Syrian) hamster model of infection provides a means of assessing nAb activity in a preclinical model of respiratory disease^16,17^. Animals inoculated with 1 × 10^5^ TCID_50_ of SARS-CoV-2 WA-1 strain intranasally were treated with STI-2020 (100, 300 or 500 µg) or STI-5041 (500 or 1000 µg) (Fig. 3) administered intravenously (IV) at 1-h post-infection. Body weight change as a percentage of starting weight, the primary clinical sign of pathogenesis in this model, was recorded and graphed (Fig. 3A,C). To control for effects of the antibody administration on animal growth, uninfected animals (UI) were administered 2,000 µg of the control IgG1 (IsoCtl) IV. Animals in this group displayed no clinical signs and gained weight throughout the course of the experiment (Supplemental Fig. 1). Hamsters infected with SARS-CoV-2 and administered 500 µg of IsoCtl-treated IV experienced steady weight loss over the first five days of infection, with maximal average weight loss reaching − 8.9% ± 2.3 at 5 days-post-infection (d.p.i.) (Fig. 3A). Intravenous administration of a 500 µg dose of STI-2020 resulted in maximum average percentage body weight loss of − 1.9%, which occurred at 2 d.p.i. Subsequently, animals treated with 500 µg of STI-2020 maintained an average body weight that as a percentage of day 0 weight was significantly different on days 3 and then throughout the study than the average weight measured among IsoCtl-treated animals. In IsoCtl-treated animals, an average of 1.6 × 10^3^ TCID_50_/g of virus was detected in lung at day 5 post-infection (Fig. 3B). Treatment with 500 µg STI-2020 resulted in reduction of virus titers below the level of detection in lungs of all animals tested, a STI-2020-treatment-related lung titer reduction of eightyfold at minimum. In the 300 µg of STI-2020 treatment group, the average virus titer in lungs was reduced below the level of detection in 3 of 5 animals tested, while 2 of 5 animals had lung titers of similar magnitude to those measured in IsoCtl-treated animals. Animals with undetectable lung virus titers in the 300 µg treatment group also experienced only moderate weight loss compared to animals with detectable lung virus titers in this group. No changes in average virus titer in lungs compared to IsoCtl- treatment were detected in animals from the 100 µg STI-2020 treatment group.

Following inoculation with 1 × 10^5^ TCID_50_ of SARS-CoV-2 Beta VOC by the intranasal route, STI-5041 was administered at either 500 µg or 1,000 µg IV at 1-h post-infection. STI-5041 protected animals from the pathogenic effects of the Beta variant. A significant difference in weight was observed at 4 d.p.i. for animals treated with 1000 µg IV STI-5041 versus IsoCtl-treated animals (1000 µg), averaging − 3.249% ± 1.738 versus − 7.534% ± 0.882, respectively (Fig. 3C). On 5 d.p.i. a significant difference in weight loss was observed in animals treated with 500 µg STI-5041 IV versus IsoCtl-treated animals (1,000 µg). Steady weight loss was observed through day 6 in Beta variant-infected animals, with the greatest percent weight loss observed on day 6 in the IsoCtl-treated (1,000 µg) group with a mean of − 12.82% ± 2.198. The maximum weight change observed in the 1,000 µg STI-5041 treated animals was at day 6 with a mean of − 4.746% ± 3.526. The maximum weight change observed in the 500 µg STI-5041 treated animals was at day 5 with a mean of − 6.857% ± 1.492. STI-5041 administered IV at either 500 or 1,000 µg resulted in significantly reduced lung virus titers as compared to infected, IsoCtl-treated animals (Fig. 3D).

### Treatment using IN administered STI-2020 or STI-5041 in the golden (Syrian) Hamster model of COVID-19

Based on the observed kinetics of STI-2020 exposure in the lungs following IN dosing at 0.5 mg/kg in mice and considering the protective efficacy of the 500 µg IV dose in the golden (Syrian) hamster model of COVID-19, we selected 500 µg as the IN dose to be administered 12 h post-infection in this hamster SARS-CoV-2 disease model. We directly compared the degree of disease severity and duration of disease in animals receiving a 500 µg dose of STI-2020 or 500 µg IsoCtl by the IN route. Animals were infected with 5 × 10^4^ TCID_50_ of SARS-CoV-2 WA-1 strain intranasally and subsequently treated with STI-2020 administered intranasally at 12 h post-infection. Weight change as a percentage of starting weight was recorded daily for each animal.

**Figure 4:**
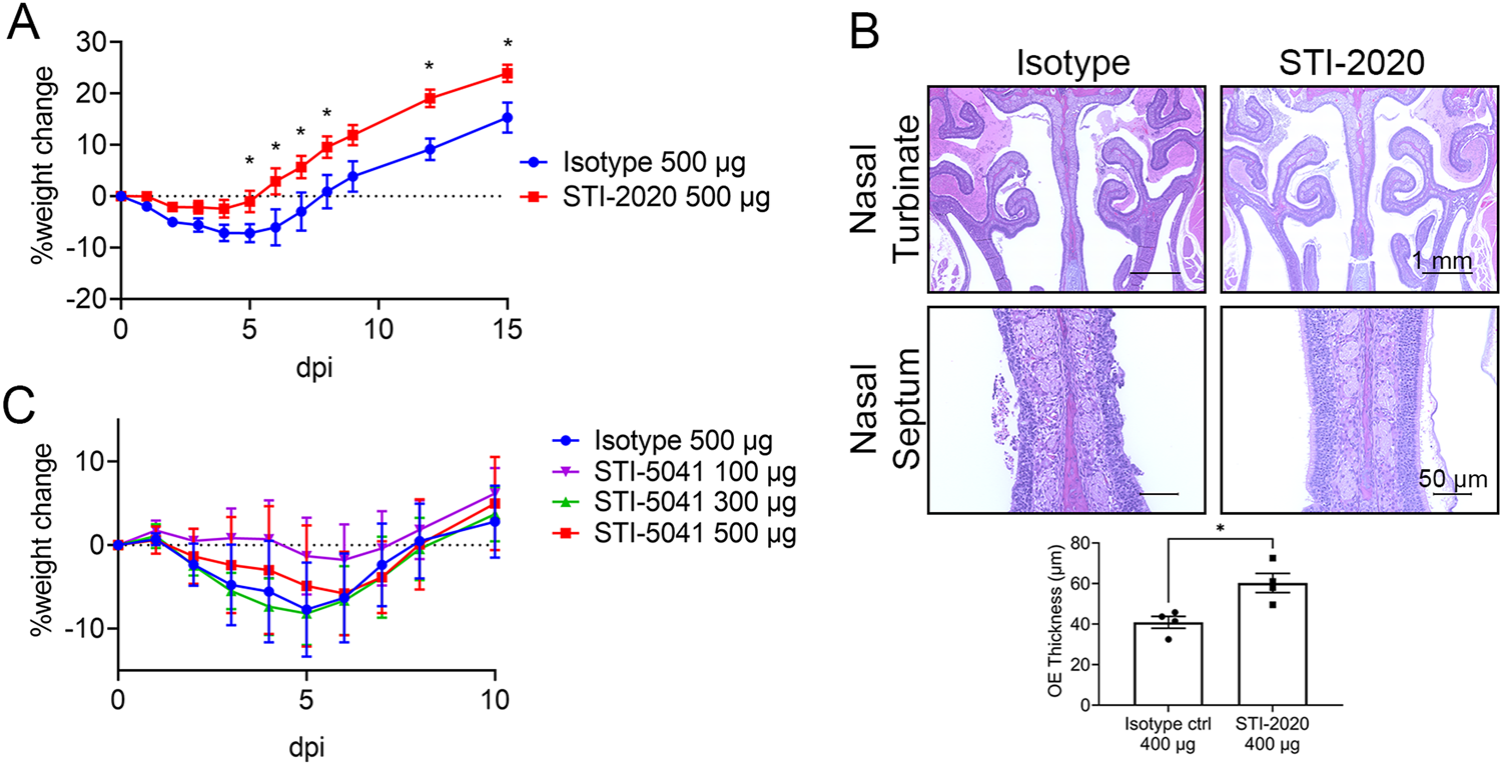
Efficacy of Intranasal (IN) Delivery of Neutralizing Antibodies in the golden (Syrian) Hamster Model of COVID-19. (**A**,**B**) Female hamsters were inoculated with SARS-CoV-2 USA/WA-1/2020, and then administrated with 500 µg or 400 µg Isotype control antibody or 500 µg or 400 µg STI-2020 intranasally at 12 h post-infection. (**A**) Average % weight change ± SEM was plotted for each group. Days in which there was a significant difference in average % weight change for STI-5020 at 500 µg compared to Isotype control IgG 500 µg-treated animals are denoted by * (*p*-value < 0.05). (**B**) Upper panels show representative figures of nasal turbinates and nasal septum at 5 d.p.i. Average ± SEM of OE thickness on the nasal septum in the lower graph for STI-5020 at 400 µg compared to Isotype control IgG 400 µg-treated animals are denoted with * (*p*-value < 0.05). (**C**) Hamsters were inoculated with SARS-CoV-2 Beta variant, and then administrated with 500 µg Isotype control antibody or 100 µg, 300 µg, or 500 µg STI-5041 intranasally at 12 h post-infection. Average % weight change ± SEM was plotted for each group.

Animals treated IN with STI-2020 at 500 µg had a maximum average weight loss of − 2.444% ± 1.714 of starting weight (4 d.p.i.) as compared to a maximum average weight loss level of − 7.194% ± 1.758 recorded in IN IsoCtl-treated animals (5 d.p.i.). The limited weight loss observed in the STI-2020-treated animals constitutes a therapeutic effect of the IN-administered antibody on the severity of COVID-19-like disease in this model. STI-2020-treated animals at 500 µg maintained their average weight (average 2.0% weight loss respectively) over the first four days of infection before steadily gaining weight, while IsoCtl-treated animals steadily lost weight across this timespan through day 5. Beginning on day 5 of infection, animals treated with 500 µg STI-2020 IN had a significant weight increase in comparison to IsoCtl-treated animals, with significant increases in weight also observed at 6, 7, 8, 12 and 15 d.p.i. (Fig. 4A).

In 400 µg STI-2020 treated IN hamsters, the olfactory epithelium (OE) thickness in the nasal turbinate septal area was measured as a readout for COVID-19-induced nasal damage^18^. There was a significant increase in OE thickness in 400 µg STI-2020 treated animals as compared to IsoCtl-treated hamsters infected with SARS-CoV-2, suggesting STI-2020 could reduce the risk for anosmia after COVID-19 infection (Fig. 4B).

To test STI-5041, 5 × 10^4^ TCID_50_ of the SARS-CoV-2 Beta variant was administered to female hamsters intranasally, 12 h post-infection STI-5041 was administered IN at 500, 300 or 100 µg, and IsoCtl was administered at 500 µg. In IsoCtl-treated animals, the greatest % weight change was observed on day 5 with a change of − 7.7% ± 2.5. At 500 µg STI-5041 IN, the greatest percent weight change was on day 6 (− 5.8% ± 2.24); 300 µg STI-5041 IN, the change in weight was day 5 (− 8.2% ± 1.7); and changes in weight were observed on day 6 with a change in weight of − 1.77% ± 1.9 for 100 µg STI-5041 IN (Fig. 4C). The therapeutic effects following treatment with IN-administered STI-5041 were maximized at the 100 ug dose level and, at higher doses, the treatment did not provide benefit beyond that observed in IsoCtl-treated animal.

## Discussion

In summary, we describe the use of the G-MAB library for the rapid identification of SARS-CoV-2 neutralizing antibodies, demonstrating its potential for developing countermeasures against any potential pathogen. Screening for G-MAB nAbs requires neither animal immunization nor availability of B-cell donor survivors. We demonstrate this with the presented screen for nAbs with pan-specific activity against SARS-CoV-2 and VOCs using an unbiased approach. Once a candidate nAb has been identified, the LALA Fc modification can be incorporated to reduce the risk for ADE. The full length human IgG1-LALA Fc antibody candidates, STI-2020 and STI-5041, demonstrated potent neutralizing activity against SARS-CoV-2, as well as relevant VOCs Alpha and Beta. In PK and biodistribution studies, IV and IN delivery of STI-2020 and STI-5041 suggests that their use in treatment of COVID-19 might be well tolerated. STI-2020 provided neutralizing protection against virus isolates and virus pseudotypes bearing amino acid changes within Spike corresponding to 2020001 and Alpha but little effect on the Beta VOC. In contrast, STI-5041 demonstrated efficacy against 2020001, Alpha and Beta, demonstrating the utility of a combination treatment as a potential future therapy. The relative half-life of both STI-2020 and STI-5041 in the lungs when administered IN suggest in the early events of infection nAb administration may prevent viral pathogenesis in the wake of diagnosis in early-stage disease. A combination of nAbs that provides a broad spectrum of VOC coverage should provide a potent means to treat early asymptomatic and mildly symptomatic COVID-19 patients, regardless of the variant causing infection.

In a hamster model of COVID-19, both STI-2020 and STI-5041 when administered intravenously and intranasally, demonstrated efficacy, reduced weight loss associated with SARS-CoV-2 infection when compared to IsoCtl-treated hamsters. When administered intranasally, STI-2020 significantly reduced weight loss and increased olfactory epithelial thickness in comparison to isotype in inoculated hamsters, raising the hypothesis that STI-2020 through viral neutralization may either promote cellular repair in the olfactory system or reduce hyperinflammation, culminating in reduced pathogenesis from COVID-19. The ability to administer nAbs both intravenously and intranasally may have important therapeutic implications, as multiple modes of effective treatment delivery could ease the therapeutic relationship between provider and patient in the setting of respiratory infection.

Based off these strong preclinical data, Phase 1 healthy subjects studies have been completed for either intravenous or intranasal administration of STI-2020, where the intranasal formulation of STI-2020 is termed STI-2099. The antibodies STI-2020 and STI-2099 have been well tolerated at the concentrations tested, with no dose limiting toxicity, serious or severe adverse events (AEs), and no AEs resulting in early termination from participation. Phase 2 studies have begun with asymptomatic or mild COVID-19 infection (clinincaltrials.gov, NCT04900428). As SARS-CoV-2 VOCs continue to emerge despite ongoing vaccination strategies, the identification of safe and effective therapies to treat the original SARS-CoV-2 strain as well as its variants remains essential.

## Materials and methods

### Antibody characterization

Kinetic interactions between the antibodies and his-tagged antigen proteins were measured at room temperature using Biacore T200 surface plasmon resonance (GE Healthcare). Anti-human fragment crystallizable region (Fc region) antibody was immobilized on a CM5 sensor chip to approximately 8,000 resonance units (RU) using standard N‑hydroxysuccinimide/N‑Ethyl-N′-(3-dimethylaminopropyl) carbodiimide hydrochloride (NHS/EDC) coupling methodology. The antibody (1.5 μg/mL) was captured for 60 s at a flow rate of 10 μL/min. The SARS-CoV-2 Spike S1, SARS-CoV-2 (2019-nCoV) Spike S1- B.1.1.7 lineage mut (HV69-70 deletion, Y144 deletion, N501Y, A570D, D614G, P681H)-His and SARS-CoV-2 (2019-nCoV) Spike S1- B.1.351 lineage mut (K417N, E484K, N501Y, D614G)-His proteins were run at six different dilutions in a running buffer of 0.01 M HEPES pH 7.4, 0.15 M NaCl, 3 mM EDTA, 0.05% v/v Surfactant P20 (HBS‑EP+). All measurements were conducted in HBS-EP+ buffer with a flow rate of 30 μL/minute. The affinity of antibody was analyzed with BIAcore T200 Evaluation software 3.1. A 1:1 (Langmuir) binding model is used to fit the data.

### Cell based spike binding assay

Mammalian expression vectors were constructed by cloning of the synthesized gene fragments encoding SARS-CoV-2 Spike variant proteins (see attached table indicating mutations introduced into wild type [WA-1 strain] spike protein sequence). HEK293 cells were transfected using FuGeneHD transfection reagent according to manufacturer’s protocol (Promega, Cat # E2311). 48 h post-transfection, cells were harvested using enzyme free cell dissociation buffer (ThermoFisher, Cat #13151014.), washed once and resuspended in FACS buffer (DPBS + 2% FBS) at 2 × 10^6^ cells/mL. For antibody binding to the cells expressing the Spike proteins, the cells were dispensed into wells of a 96-well V bottom plate (40 µL per well), and an equal volume of 2 × final concentration of serially-diluted anti-S1 antibody solution was added. After incubation on ice for 45 min, the cells were washed with 2 times of 150 µL FACS buffer. Detection of bound antibody was carried out by staining the cells with 50 µL of 1:500 diluted APC AffiniPure F(ab')_2_ Fragment (Goat Anti-Human IgG (H + L). Jackson ImmunoResearch, Cat# 109-136-4098) for 20 min on ice. The cells were washed once with 150 µL FACS buffer and analyzed on IntelliCyt iQue® Screener (Sartorius) flow cytometry. Mean fluorescent intensity values were obtained from the histograms. A sigmoidal four-parameter logistic equation was used for fitting the MFI versus. mAb concentration data set to extract EC50 values (GraphPad Prism 8.3.0 software).

### Cells and viruses

Vero E6 cells were maintained in Dulbecco’s modified Eagle’s medium (DMEM, Corning, NY) supplemented with 10% fetal bovine serum (FBS, Thermo Fisher Scientific, MA), 1% penicillin–streptomycin, and L-glutamine. The P3 stock of the SARS-CoV-2 USA/WA-1/2020, 202001, USA/CA-CDC5574/2020 and, MD-HP01542/2021 isolates were obtained from The World Reference Center for Emerging Viruses and Arboviruses (WRCEVA) at the University of Texas Medical Branch. The viruses were propagated in Vero E6 cells and cell culture supernatant of P4 stocks were stored at − 80 °C under BSL3 conditions.

### SARS-CoV-2 neutralization assay

The day before infection, 2 × 10^4^ Vero E6 cells were plated to 96-well plates and incubated at 37° C, 5% CO_2_. Monoclonal antibodies were twofold serially diluted in infection media (DMEM + 2%FBS). Sixty microliters of diluted samples were incubated with 200 µL 50% tissue culture infective doses (TCID_50_) of SARS-CoV-2 in 60 µL for 1 h at 37 °C. One-hundred microliters of the antibody/virus mixture were subsequently used to infect monolayers of Vero E6 cells grown on 96-well plates. Cells were fixed with 10% formalin and stained with 0.25% crystal violet to visualize cytopathic effect (CPE). The neutralizing concentrations of monoclonal antibodies were determined by complete prevention of CPE.

### Plasmids

All SARS-CoV-2 Spike constructs for pseudotype generation were expressed from plasmid pCDNA3.1 (Thermo Fisher, Cat #V79020). For the pseudovirus neutralization study, codon optimized SARS-CoV-2 Wuhan Spike carrying the D614G amino acid change (Sino Biological, Cat #VG40589-UT(D614G)) was modified to remove the last 21 amino acids at the C-terminus (SpikeΔ21) and was used as the parental clone. For the Alpha variant, amino acid changes included Δ69–70, Δ144, N501Y, A570D, D614G, P681H, T716I, S982A, and D1118H. For the Beta variant, amino acid changes included D80A, D215G, Δ242–244, K417N, E484K, N501Y, D614G, and A701V. For the HEK293 expressed spike variant study, the codon optimized genes encoding full-length spike proteins.

**Table:** 

Variant	Mutations (and mutations in RBD from 333 to 526)
Alpha (B1.1.7) variant	deletion of 69–70, deletion of Y144, **N501Y**, A570D, D614G, P681H, T716I, S982A, D1118H
Beta (B1.351) variant	L18F, D80A, D215G, deletion of 242–244, R246I, **K417N**, **E484K**, **N501Y**, D614G, A701V

### Cells and media

BHK21 cells (ATCC, Cat #CCL-10) were maintained in DMEM/F12 media (Thermo Fisher, Cat #21041025) supplemented with 10% fetal bovine serum (Omega Scientific, Cat #FB-02) and 5% trypose phosphate broth (Thermo Fisher, Cat #18050039). BHK21/WI-2 cells (Kerafast, Cat #EH1011) were maintained in DMEM (Thermo Fisher, Cat #11965092) supplemented with 5% fetal bovine serum. 293-ACE2 cells were maintained in DMEM supplemented with 10% fetal bovine serum and 200 µg/mL G418 (Invivogen, Cat #ant-gn-2).

### VSV-Spike pseudotype generation

To generate each Spike pseudotyped VSV, 1.2E6 BHK21 cells were nucleofected with 2 µg of Spike plasmid using an Amaxa Nucleofector II with cell line kit L (Lonza, Cat #VCA-1005) and program A-031. Cells were plated to one well of a 6-well dish and incubated overnight at 37 °C/5%CO_2_. The next day, cells were transduced with Pseudotyped ΔG-luciferase (G*ΔG-luciferase) rVSV (Kerafast, Cat #EH1025-PM) at MOI ~ 4 for 1 h at 37 °C/5%CO_2_. Cells were rinsed twice with DPBS (Corning, Cat #21-031-CM), 2 mL of fresh media added, and incubated for 24–44 h at 37 °C/5%CO_2_. Supernatants were collected, spun at 300xg for 5 min at room temperature, aliquoted and stored at − 80 °C. Pseudotypes were normalized for luciferase expression by incubating with 1 µg/mL anti-VSV-G clone 8G5F11 (Millipore, Cat #MABF2337) for 30 min at room temperature followed by transduction of 293-ACE2 cells. G*ΔG-luciferase VSV of known titer was used as the standard. Transduced cells were incubated for 24 h and luminescence measured using a Tecan Spark plate reader.

### Neutralization assays

293-ACE2 cells were plated into 96-well white plates at 40,000 cells/well and incubated at 37 °C/5% CO_2_. The next day, pseudotyped VSV was incubated with anti-spike (concentration as indicated) and anti-VSV-G (1 µg/mL) antibodies for 30 min at room temperature and added to the 293-ACE2 cells in triplicate. Transduced cells were incubated for 24 h and luminescence measured using a Tecan Spark plate reader. The percent inhibition was calculated using 1 − ([luminescence of antibody treated sample]/[average luminescence of untreated samples]) × 100. Absolute IC50 was calculated using non-linear regression with constraints of 100 (top) and 0 (baseline) using GraphPad Prism software. Data represents results from at least 3 independent experiments, each with samples in triplicate. Negative value slopes were assigned IC50 of > 10 µg/mL.

### Affinity measurements

Kinetic interactions between the antibodies and his-tagged antigen proteins were measured at room temperature using Biacore T200 surface plasmon resonance (GE Healthcare). Anti-human fragment crystallizable region (Fc region) antibody was immobilized on a CM5 sensor chip to approximately 8,000 resonance units (RU) using standard N‑hydroxysuccinimide/N‑Ethyl-N′-(3-dimethylaminopropyl) carbodiimide hydrochloride (NHS/EDC) coupling methodology. The antibody (1.5 μg/mL) was captured for 60 s at a flow rate of 10 μL/minute. The SARS-CoV-2 Spike S1, SARS-CoV-2 (2019-nCoV) Spike S1- B.1.1.7 lineage mut (HV69-70 deletion, Y144 deletion, N501Y, A570D, D614G, P681H)-His and SARS-CoV-2 (2019-nCoV) Spike S1- B.1.351 lineage mut (K417N, E484K, N501Y, D614G)-His proteins were run at six different dilutions in a running buffer of 0.01 M HEPES pH 7.4, 0.15 M NaCl, 3 mM EDTA, 0.05% v/v Surfactant P20 (HBS‑EP +). All measurements were conducted in HBS-EP + buffer with a flow rate of 30 μL/min. The affinity of antibody was analyzed with BIAcore T200 Evaluation software 3.1. A 1:1 (Langmuir) binding model was used to fit the data.

### Epitope binning

Epitope binning of STI-2020 and STI-5041 SARS-CoV-2 S1 was performed by BLI using an Octet RED96 instrument. His-tagged SARS-CoV-2 S1 was captured onto NTA biosensors and coated with STI-2020 at a saturating concentration of 10 µg/mL for 240 s. The epitopes of STI-5041 were probed in relation to STI-2020 by assaying the STI-2020-coated biosensors in 10 µg/mL of STI-5041 together with same concentration of STI-2020. All graphs were overlaid and aligned at the baseline after S1 capture.

### Biodistribution study

Female CD-1-IGS mice (strain code #022) were obtained from Charles River at 6–8 weeks of age. For intravenous injection of STI-2020, 100 µL of antibody diluted in 1X HBSS was administered retro-orbitally to anesthetized animals. For intranasal instillations, antibody was diluted in 1X HBSS and administered by inhalation into the nose of anesthetized animals in a total volume of 20 µL (10 µL per nostril) using a pipette tip. Organs, blood, and lung lavage samples were collected 24 h post-antibody administration. Blood was collected by retro-orbital bleeding and then transferred to Microvette 200 Z-Gel tubes (SARSTEDT, Cat No# 20.1291, lot# 8071211). Tubes were then centrifuged at 10,000* g* for 5 min at room temperature. Serum was transferred into 1.5 mL tubes and stored at − 80 °C. Lung lavage samples were collected following insertion of a 20G × 1-inch catheter (Angiocath Autoguard from Becton Dickinson, Cat # 381702, lot# 6063946) into the trachea. A volume of 0.8 mL of PBS was drawn into a syringe, placed into the open end of the catheter, and slowly injected and aspirated 4 times. The syringe was removed from the catheter, and the recovered lavage fluid was transferred into 1.5 mL tubes and kept on ice. Lavage samples were centrifuged at 800* g* for 10 min at 4 °C. Supernatants were collected, transferred to fresh 1.5 mL tubes, and stored at − 80 °C. Total spleen, total large intestine, and 150 to 400 mg of lungs and small intestine were suspended in 300 µL of PBS in pre-filled 2.0 mL tubes containing zirconium beads (Spectrum, Cat # 155-40945). Tubes were processed in a BeadBug-6 homogenizer at a speed setting of 3,000* g* and a 30 s cycle time for four cycles with a 30-s break after each cycle. Tissue homogenates were centrifuged at 15,000 rpm for 15 min at 4 °C. Homogenate supernatants were then transferred into 1.5 mL tubes and stored at − 80 °C. STI-2020 antibody levels in each sample were quantified using the antibody detection ELISA method. Statistical significance was determined a two-way unpaired t-test and a Welch’s correction was used when the sample size was different between groups. Experiments were conducted and reported in accordance with the ARRIVE guidelines. For intranasal instillation, mice were briefly anesthetized using a low dose isoflurane inhalation. Euthanasia was performed using a CO_2_ (30–70% vol/min, 5.7–13.3 L/min) or isoflurane inhalation (4%), followed by cervical dislocation under anesthesia. This study was reviewed and accepted by the animal study review committee (SRC) and conducted in accordance with Institutional Animal Care and Use Committee (IACUC) guidelines.

### Pharmacokinetic study

Female CD-1-IGS mice (strain code #022) were obtained from Charles River Laboratories at 6–8 weeks of age. STI-2020 and STI-5041 dissolved in intranasal formulation buffer were administered as previously described for the IN biodistribution study. Lungs and blood were collected from 3 mice at each of the following timepoints: 10 min, 1.5 h, 6 h, 24 h, 72 h, 96 h, 168 h, 240 h, and 336 h. Serum and lung tissue samples were collected as described for the biodistribution study. STI-2020 and STI-5041 antibody levels in each sample were quantified using the antibody detection ELISA method. Briefly, 96-well plates were coated with mouse anti-human CH2 specific IgG antibody at 2 μg/mL in PBS 1X (50 μL/well), covered with plate sealer and kept overnight at 4 °C. Plates were washed three times with 1X washing solution (KPL wash solution) and blocked with 50 μL/well of Blocker™ Casein in PBS for 1 h at room temperature on shaker at a rate of 4–5. Plates were washed three times with 1X washing solution. 50 μL/well of lungs tissue lysates (diluted 1:250 in Blocker™ Casein in PBS) or serum (diluted 1:150 in Blocker™ Casein in PBS) were transferred to the plates. To generate a standard curve, cognate IgG1[LALA] monoclonal antibody was serially diluted (from 1,000 ng/mL to 1.95 ng/mL) in Blocker™ Casein in PBS and 50 μL/well added on the same plate. The plates were incubated for 2 h at room temperature on a shaker. Plates were washed three times with 1X washing solution. 50 μL/well of Sulfo-Tag anti-human/NHP IgG antibody (Meso Scale Discovery, Cat# D20JL-6) (diluted at 1:1,000 in Blocker™ Casein in PBS) were transferred to the plates and incubated for 1.5 h at room temperature on a shaker at a rate of 4–5. Plates were washed three times with 1X washing solution. To detect the presence of STI-2020, 150 μL/well of 2X read Buffer were dispensed into the wells of the 96-well plates. Plates were read immediately on a MSD instrument (Meso Sector S600).The standard curve (linear range of assay between 250 and 1.95 ng/mL for STI-2020 and between 125 and 7.81 ng/ml for STI-5041) generated with cognate IgG1[LALA] monoclonal antibodies for STI-2020 and STI-5041 were used to determine the concentration of antibody in both lung lysates and serum. Pharmacokinetic analysis of the collected ELISA data was performed with the Phoenix WinNonlin software (Certara, version 6.4) using a non-compartmental approach consistent with an IN bolus route of administration. Statistical significance was determined using a two-way unpaired t-test and a Welch’s correction was used when the sample size was different between groups. Experiments were conducted and reported in accordance with the ARRIVE guidelines. For intranasal instillation, mice were briefly anesthetized using a low dose isoflurane inhalation. Euthanasia was performed using a CO_2_ (30–70% vol/min, 5.7–13.3 L/min) or isoflurane inhalation (4%), followed by cervical dislocation under anesthesia. This study was reviewed and accepted by the animal study review committee (SRC) and conducted in accordance with IACUC guidelines.

### Hamster challenge experiments

Female golden (Syrian) hamsters were obtained from Charles River Laboratories at 6 weeks of age. Hamsters were inoculated intranasally (i.n.) with 10^5^ TCID_50_ of SARS-CoV-2 in 100 µL of sterile PBS on day 0. Antibody treatments were administered intravenously (i.v.) with monoclonal antibodies (mAbs) against SARS-CoV-2 Spike, or isotype control mAb in up to 500 µL of sterile PBS at 1 h-post inoculation. For intranasal instillation of antibodies, 100 µL of formulated material was introduced directly into the nostrils and inhaled by anesthetized animals. Animals were monitored for illness and mortality for up to 12 days post-inoculation and clinical observations were recorded daily. Body weights and temperatures were recorded at least once every 72 h throughout the experiment. On day 5 post-infection, 5 animals from each treatment group were euthanized and lung samples were collected for virus. Average % weight change on each experimental day was compared with the isotype control mAb-treated group using a two-way ANOVA following Dunnett’s multiple comparisons test. All animals were housed in animal biosafety level-2 (ABSL-2) and ABSL-3 facilities in Galveston National Laboratory at the University of Texas Medical Branch. Experiments were conducted and reported in accordance with the ARRIVE guidelines. Isoflurane inhalation was used for anesthesia. A high-flow rate of CO_2_ followed by thoracotomy were used at the time of euthanasia. All animal studies were reviewed and approved by the IACUC at the University of Texas Medical Branch and were conducted according to the National Institutes of Health guidelines.

### Determination of infectious virus titers in the lung

Prior to initiation of the experiment, five animals from each treatment group (n = 10) were designated for virus titration in lung tissue at 5 days post inoculation. On the assigned day, animals were euthanized, lung tissue samples were collected from each animal, and a portion of the tissue (0.08–0.3 g) was placed into pre-labeled microcentrifuge tubes containing 5 mm stainless steel beads (Qiagen Inc., CA). Lung samples were homogenized with DMEM + 2% FBS in a TissueLyser (Qiagen Inc., CA) operated at 25–30 Hz for four minutes. Tubes were centrifuged and clarified homogenate was serially diluted tenfold with DMEM + 2% FBS. From material representing each serial dilution step, 100 µL was transferred to each of four wells of a 96-well plate previously seeded with Vero E6 cells. Plates were then incubated for 72–96 h at 37 °C, 5% CO_2_. Cells were subsequently fixed with 10% formalin and stained with 0.25% crystal violet solution. TCID_50_ values were calculated by the method of Reed and Muench^19^. Virus titers in lungs were compared with the isotype control mAb-treated group using one-way ANOVA following Dunnett’s multiple comparisons test.

### Histopathology of nasal turbinates from hamster model

Histopathology analyses of nasal turbinates were conducted as previously reported^18^. In brief, nasal turbinates were collected at the time of euthanasia and fixed with 10% formalin for 7 days before removal from the ABSL-3 facility. Nasal turbinates samples were decalcified with EDTA (10% w/v) and embedded in paraffin prior to sectioning. Sections were 5 μm thick, and subsequently were stained with Hematoxylin and Eosin. The OE thickness of the nasal septum was measured using light microscopy. OE thickness of STI-2020 treated hamsters were compared to isotype-control treated hamsters using an unpaired t-test.

## Supplementary Information

Supplementary Information.

## Data Availability

Data underlying the study will be made available upon request to Henry Ji.
