# Supplementary material for: Unbiased approach to identify and assess efficacy of human SARS-CoV-2 neutralizing antibodies
Source: Sci Rep. 2022 Sep 15;12:15517. doi: 10.1038/s41598-022-19780-7 (PMC9476467; doi:10.1038/s41598-022-19780-7)

Supplementary Materials for

Unbiased Approach to Identify and Assess Efficacy of Human SARS-CoV-2 Neutralizing Antibodies

**AUTHORS**

Xia Cao^1^, Junki Maruyama^2^, Heyue Zhou^1^, Yanwen Fu^1^, Lisa Kerwin^1^, Colin Powers^1^, Rachel A. Sattler^2^, John T. Manning^2^, Alok Singh^1^, Reyna Lim^1^, Laura D. Healy^1^, Sachi Johnson^1^, Elizabeth Paz Cabral^1^, Donghui Li^1^, Arthur Ledesma^1^, Daniel Lee^1^, Susan Richards^1^, Laura Rivero-Nava^1^, Yan Li^1^, Weiqun Shen^1^, Karen Stegman^1^, Benjamin Blair^1^, Shinji Urata^2^, Magumi Kishimoto-Urata^2^, Jamie Ko^1^, Na Du^1^, Kyndal Morais^1^, Kate Lawrence^1^, Ianne Rivera^1^, Lucy Lu^1^, Chin-I Pai^1^, Damien Bresson^1^, Mark Brunswick^1^, Yanliang Zhang^1^, Henry Ji^1*^, Slobodan Paessler^2*^, Robert D. Allen^1^

Fig. S1.

**Intravenous isotype control antibody weight change data of uninfected hamsters.** Female hamsters were administrated 2000 µg Isotype control antibody. Average % weight change ± SEM was plotted.


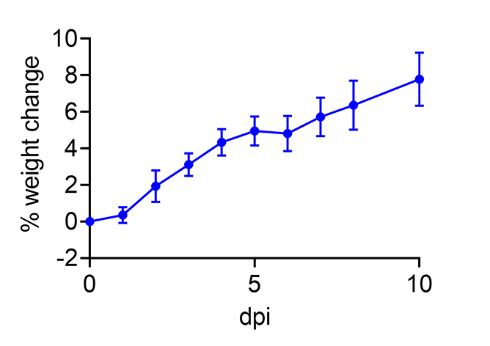

Supplement: Supplementary file 1 — Supplementary Information. [file 41598_2022_19780_MOESM1_ESM.docx]
